# QTL mapping of male sterility and transmission pattern in progeny of Satsuma mandarin

**DOI:** 10.1371/journal.pone.0200844

**Published:** 2018-07-17

**Authors:** Shingo Goto, Terutaka Yoshioka, Satoshi Ohta, Masayuki Kita, Hiroko Hamada, Tokurou Shimizu

**Affiliations:** Division of Citrus Research, Institute of Fruit Tree and Tea Science, NARO, Shizuoka, Japan; USDA-ARS Southern Regional Research Center, UNITED STATES

## Abstract

Seedlessness is one of the important traits in citrus breeding. Male sterility derived from Satsuma mandarin (*Citrus unshiu*) has been used in Japanese citrus breeding programs to obtain seedless cultivars. The efficiency of seedless cultivar breeding would be improved by developing a selection marker linked to seedlessness. In this study, we performed QTL mapping in ‘Okitsu No. 46’ × ‘Okitsu No. 56’ (O46-O56) crosses for the number of pollen grains per anther (NPG) and apparent pollen fertility (APF), two traits used as an index of male sterility, and detected a candidate QTL for NPG (*MS-P1*) on linkage group 8 with a significant LOD score (7.31) and 47% of variance explained. The QTL for APF (*MS-F1*) was detected on linkage group 6 with a significant LOD score (5.71) and 63.6% of variance explained. The role of both *MS-P1* in reducing NPG and *MS-F1* in decreasing APF were confirmed with the ‘Okitsu No.46’ × ‘Kara’ (O46-K) cross. Pedigree analysis inferred that both *MS-P1* and *MS-F1* in ‘Okitsu No. 46’ were derived from kunenbo (*Citrus nobilis*) through hassaku (*C*. *hassaku*) and ‘Sweet Spring’. Cytoplasm analysis revealed that both male-sterile ‘Sweet Spring’ and ‘Okitsu No. 46’ have cytoplasm derived from Kishu (*C*. *kinokuni* hort. ex Tanaka), but the cytoplasm of male-sterile kunenbo and hassaku were derived from other varieties rather than Kishu. These results suggest that *MS-P1* and *MS-F1* primarily reduce the NPG and decrease APF, but their expression requires a cytoplasm derived from Kishu. These findings will improve our understanding of the molecular mechanism of male sterility in citrus and help to develop a DNA marker for seedless breeding in citrus.

## Introduction

Satsuma (*Citrus unshiu* Marcov.) is the most produced citrus cultivar in Japan and has been cultivated in other countries. One of the favorable traits in Satsuma is its seedlessness, an important trait in citrus breeding programs. Seedlessness in Satsuma is caused by a combination of male sterility, female sterility, and parthenocarpy [1,2]. Especially, male sterility derived from Satsuma has been used in Japanese citrus breeding programs to select for seedless varieties [3]. Recent studies have proposed that male sterility derived from Satsuma is caused by the cooperative action of the cytoplasm derived from Satsuma and nuclear genes [4–6]. Especially, Yamamoto et al. suggested the cooperative action by reciprocal crosses [5], and Guo and his colleagues proposed that the mitochondrial genome or its interaction with nuclear genes is involved in the control of cytoplasmic male sterility [7]. Efforts have been made to introduce male sterility of Satsuma to other varieties by transferring Satsuma’s cytoplasm by cybrid formation [8–10]. A male sterile cybrid line G1+HBP, whose nuclear genome and chloroplast DNA are inherited from the leaf parent Hirado Buntan pummelo (*C*. *grandis* [L.] Osbeck) (HBP) and mitochondrial genome from the callus parent Satsuma cultivar Guoqing No. 1 (G1), was regenerated [8] and confirmed to be male-sterile and seedless with a severe degeneration of stamen and petals [11,12] and aborted pollen grains in anthers [13]. In contrast, our recent study indicated that both the number of pollen grains per anther (NPG) and the apparent pollen fertility (APF) of male-sterile varieties, which are used to compute the index of male sterility, are inherited by their progeny and suggested the involvement of a nuclear factor [14]. This study also suggested that the cooperative action of the cytoplasm derived from Satsuma and the nuclear gene(s) is a prerequisite for the expression of NPG [14]. However, it remains to be clarified whether nuclear genes are involved in male sterility derived from Satsuma.

Conventional citrus breeding by cross-hybridization has been performed since 1937 in Japan [15]. Breeding of new citrus varieties is time-consuming and costly as the large size of citrus trees limits the number of cross seedlings in the field that can be implemented for selection. Furthermore, the seedlings usually take at least five years to bloom and require a long-term maintenance before they can be selected as cultivar candidates. These problems can be solved by developing selection markers by QTL analysis and their application in marker-assisted selection (MAS) at the seedling stage [16,17]. QTL analysis and MAS facilitate the selection of seedlings with superior traits indirectly by selecting haplotype blocks harboring QTL by using flanking markers. The success of MAS primarily depends on not only the power for selecting the target trait with a DNA marker but also the availability of the DNA marker for a wide range of accessions.

Recently, Shimizu and colleagues evaluated the parentage of citrus varieties based on DNA marker analysis of 286 genotypes for nuclear and cytoplasmic genomes and inferred the parentages in 69 indigenous varieties [18]. According to their observations, the seed and pollen parent of Satsuma are Kishu (*C*. *kinokuni* hort. ex Tanaka) and kunenbo (*C*. *nobilis* Lour. var. *kunep* Tanaka), respectively. The parentage of Satsuma, kunenbo, and Kishu was further verified by parentage analysis with genome-wide SNP markers selected from whole genome sequences [19]. The genotype data provided by Shimizu et al. [18] can be utilized to deduce the transmission pattern of each allele linked to a trait of interest.

In this study, we aimed (1) to identify QTLs related to NPG and APF by analyzing F_1_ populations that show segregation for these traits; (2) to determine the association between male sterility and alleles near the QTLs; (3) to verify the availability of QTLs in other F_1_ populations that show segregation for male sterility; and (4) to infer the transmission pattern of the haplotype block containing QTLs.

## Materials and methods

### Plant materials and evaluation of male sterility

‘Okitsu No. 46’ (‘Sweet Spring’ [Satsuma ‘Ueda’ × hassaku] × sweet orange ‘Trovita’), ‘Okitsu No. 56’ (‘Okitsu No. 45’ [‘Kiyomi’ × ‘Wilking’] × ‘Nou No. 5’ [‘Lee’ × seedless Kishu]), and ‘Kara’ (Satsuma ‘Owari’ × King mandarin) were used as cross parents in this study. Two F_1_ populations of ‘Okitsu No. 46’ × ‘Okitsu No. 56’ (O46-O56) and ‘Okitsu No. 46’ × ‘Kara’ (O46-K) crosses that have been maintained at the Division of Citrus Research, Institute of Fruit Tree and Tea Science, NARO (Shizuoka, Japan), were provided for this study (S1 Table). Their seedlings were grafted onto trifoliate orange rootstock as a single replicate in April 2012. The details about grafting and cultivation were described in our previous report [14]. The NPG and APF of individual seedlings in the O46-O56 and the O46-K populations in 2014 and 2015 were evaluated previously [14,20] and those in the O46-O56 in 2016 were newly evaluated in this study (S1 Table). A protocol for evaluation of the total number of pollen grains per anther and the apparent pollen fertility used in this study is open in protocols.io (http://dx.doi.org/10.17504/protocols.io.q78dzrw).

### Genomic DNA extraction and genotyping analysis with SSR markers

Genomic DNA was extracted from fresh leaves of F_1_ populations (O46-O56 and O46-K) and their parents by a modified protocol using a Nucleon PhytoPure kit (GE Healthcare Life Science, Buckinghamshire, UK) [21]. Genotyping data of the F_1_ populations were obtained from 235 SSR primer pairs; 182 SSRs of the 235 SSRs were previously reported [18,22–24], and 53 SSRs were newly developed (S2 Table). The multiplexed and multicolored post-labeling method was applied to the genotyping analysis [25]. Data collection and allele calling were conducted as previously described [18].

### Linkage analysis and map construction

Genotype data obtained from the SSR marker analysis were translated into the cross pollinator (CP) mode format with GUGS (General Utilities for Genotyping Study) software (Shimizu, in preparation). Linkage maps were constructed for O46-O56 by using the double pseudo-testcross mapping strategy [26] with CP mode in JoinMap ver. 4.1 [27]. The markers were grouped with the independence test logarithm (base 10) of the odds (LOD), with a minimum LOD threshold of 3.0. Map distances were calculated using maximum likelihood mapping algorithm and Haldane’s map function [28].

### QTL analysis

The NPG values were square-root transformed, and APF data were arcsine-transformed because of the non-normal distribution; normal distribution of the transformed data was confirmed with the Shapiro-Wilk test [29] provided by the R stats package. The transformed data were subjected to QTL analysis with MapQTL ver. 6.0 [30]. The QTL detection was initially carried out by using the parental maps of ‘Okitsu No. 46’ and ‘Okitsu No. 56’ separately by the interval mapping (IM) method. Then, the data were reanalyzed using an integrated map to confirm the QTLs detected on the parental linkage maps. Genome-wide LOD thresholds were calculated with a 1000-permutation test for each trait on all linkage groups and identified QTLs at *p* < 0.05. Subsequently, we carried out the multiple QTL mapping (MQM) method using the SSR markers nearest to the QTL positions as cofactors on the integrated map, and QTLs were identified at *p* < 0.1. Kruskal-Wallis (KW) test was used to confirm the QTLs associated with NPG and APF.

### Inferring the transmission pattern of haplotype blocks containing QTLs

The haplotype phase of the region harboring QTLs for NPG and APF was determined for ‘Okitsu No. 46’ by using the integrated map of O46-O56. The transmission patterns of the alleles of the nearest marker to the QTLs were inferred for the ancestors of ‘Okitsu No. 46’ and ‘Kara’ by using their genotypes [18] and by pedigree-based analysis.

## Results

### Categorization of male sterility

Satsuma, ‘Okitsu No. 46’, and ‘Sweet Spring’ have been categorized as male-sterile in citrus breeding [1,6,31]. The NPG in Satsuma in 2017 was 1,320 (S1 Fig), but no pollen grains were observed on the outside of their anthers during anthesis (S2 Fig); APF was 30% (S1 Fig). ‘Okitsu No.46’ showed similar results. These observations indicate a quite low possibility of self-pollination in Satsuma and ‘Okitsu No.46’. In contrast, the NPG in ‘Okitsu No. 56’ and ‘Kara’ was approximately 5,000 (S1 Fig) and abundant yellow pollen grains were observed on the outside of their anthers (S2 Fig). In addition, APF in both cultivars was approximately 90% (S1 Fig). These results show that Satsuma and ‘Okitsu No. 46’ are male-sterile, which is in accord with previous reports [1,6], and ‘Okitsu No. 56’ and ‘Kara’ are male-fertile. We crossed ‘Okitsu No. 46’ with ‘Okitsu No. 56’ and ‘Kara’ and obtained two F_1_ populations (O46-O56 and O46-K).

### Linkage map construction of O46-O56

**Fig 1:**
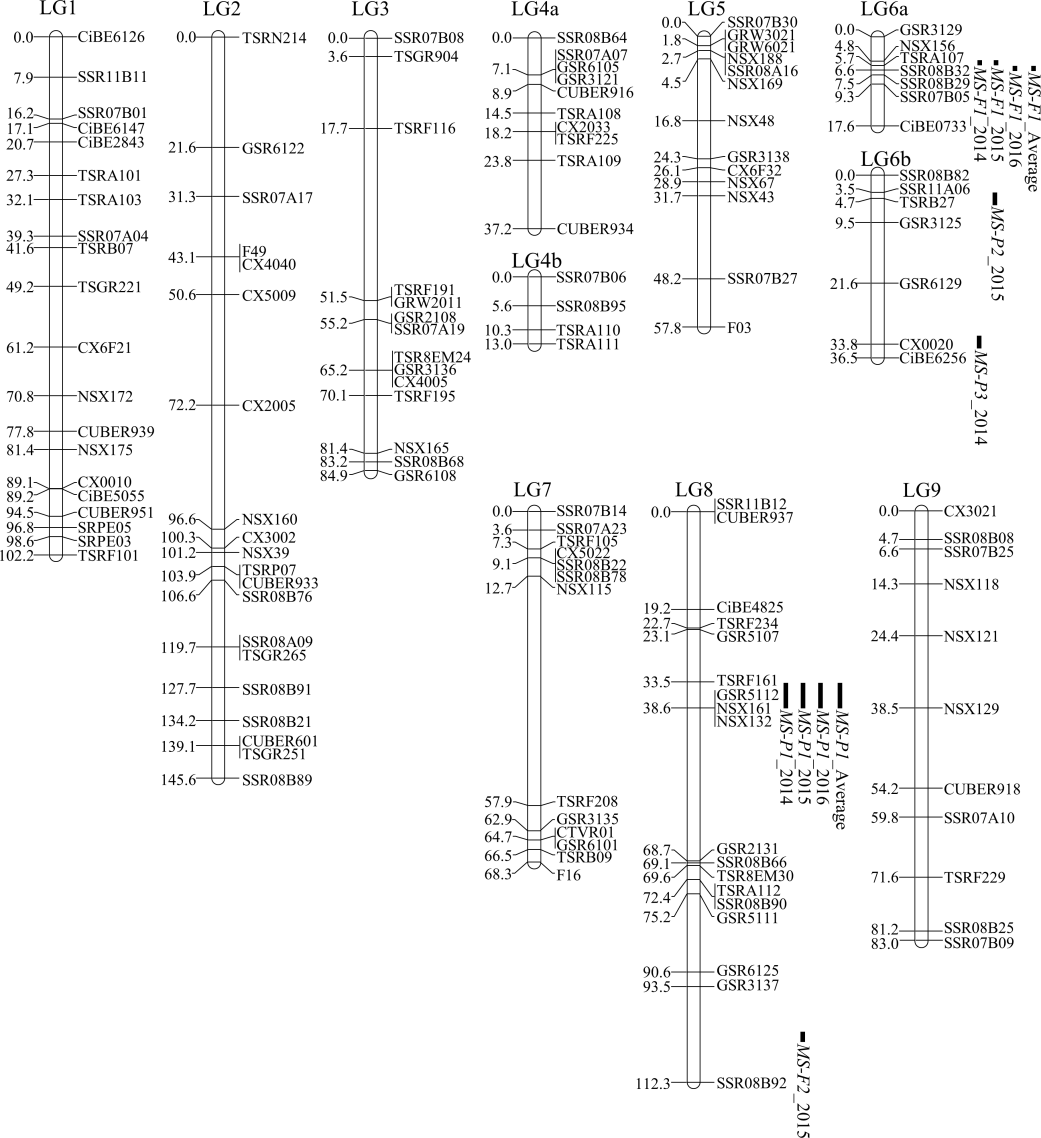
A linkage map for ‘Okitsu No. 46’ × ‘Okitsu No. 56’ population and quantitative trait loci. Marker names are on the right of each linkage group (LG), and genetic distances (cM) are on the left. The significant quantitative trait loci (QTLs) are shown on the right side of each LG, and black boxes indicate significant intervals by multiple QTL mapping analysis (*p* < 0.10). The significant intervals are determined based on evaluations conducted in 2014–2016 and the average of the three years. *MS-P1*, *2*, and *3* are QTLs for the number of pollen grains per anther. *MS-F1* and *2* are QTLs for apparent pollen fertility.

We detected <nnxnp>, <lmxll>, <hkxhk>, <efxeg>, and <abxcd> type of genotype for 235 markers in 57 seedlings of the O46-O56 population (S3 Table). Maternal and paternal linkage maps were constructed with 56 makers of <nnxnp> type and 98 makers of <lmxll> type of genotype and integrated with 81 markers that showed <hkxhk>, <efxeg>, and <abxcd> segregations. The integrated map consisted of 235 marker loci, spanning 758.5 cM on 11 linkage groups and LOD score of 3.0–7.0 (Fig 1 and S3 Table). These markers were assigned to major nine scaffolds of the reference Clementine genome sequence by referring the position of DNA markers in the Clementine genome; linkage groups (LG) 4a and LG 6a were connected to LG 4b and LG 6b [32], respectively (Fig 1).

### QTL mapping

The NPG and APF in O46-O56 population were evaluated in 34 seedlings in 2014, 49 seedlings in 2015, and 54 seedlings in 2016. Among those seedlings, 25 seedlings were common in 2014, 2015, and 2016 (2014–2016) (S1 Table). The significant QTLs for the NPG and APF were initially detected by evaluating the data obtained in 2014–2016 and then confirmed with their average by using IM analysis (S4 Table). The detected QTLs were confirmed by MQM and KW analysis (Fig 1 and Table 1).

**Table 1:** Quantitative trait loci (QTLs) for the number of pollen grains per anther (NPG) and apparent pollen fertility (APF) in ‘Okitsu No. 46’ × ‘Okitsu No. 56’ population obtained by multiple QTL mapping (MQM) for three consecutive years.

Traits	QTL	Data	LG	Position (cM)	LOD (MQM)	Significance (KW)	% Expl. (MQM)	Nearest marker
NPG	*MS-P1*	2014	8	38.6	8.39	******	56.1	GSR5112, NSX161, NSX132
		2015	8	36.5	4.86	******	24.9	
		2016	8	38.6	6.95	*******	44.7	
		Average	8	37.5	8.92	***	37.7	
	*MS-P2*	2014	-	-	n.d.	-	-	-
		2015	6b	3.5–4.7	3.64	***	18.2	SSR11A06, TSRB27
		2016	-	-	n.d.	-	-	-
		Average	-	-	n.d.	-	-	-
	*MS-P3*	2014	6b	33.8	3.89	-	18.5	CX0020
		2015	-	-	n.d.	-	-	-
		2016	-	-	n.d.	-	-	-
		Average	-	-	n.d.	-	-	-
APF	*MS-F1*	2014	6a	5.7	4.79	*****	47.7	TSRA107
		2015	6a	5.7	7.01	*******	36.2	
		2016	6a	6.6	4.27	******	30.5	SSR08B32
		Average	6a	6.6	4.12	*****	53.1	
	*MS-F2*	2014	-	-	n.d.	-	-	-
		2015	8	102.5	3.51	****	14.6	GSR3137
		2016	-	-	n.d.	-	-	-
		Average	-	-	n.d.	-	-	-

QTL: highest logarithm of the odds (LOD) value. LG: linkage group. Position: locus with the highest LOD. % Expl.: the percentage values of phenotypic variation explained by QTL. Nearest marker: the nearest maker to QTL. Average: the average value obtained for 2014–2016. All the data were analyzed by using the Kruskal-Wallis (KW) test module to confirm the detected QTLs because of their non-normal distribution. n.d.: indicates that significant LOD value was not detected. Significance: asterisks (***, ****, *****, ******, *******) show significance levels at *p* < 0.01, < 0.005, < 0.001, < 0.0005, and < 0.0001, respectively.

The IM analysis revealed significant QTLs at *p* = 0.05 for NPG at *MS-P1* on LG8 (2014–2016 and the average for the three years) and *MS-P2* on LG6b (2015) (S4 Table). In addition, the MQM analysis of those loci confirmed significant QTLs at *p* = 0.1 at *MS-P1* on LG8 (2014–2016 and the average for the three years) and *MS-P2* on LG6b (2015) and detected *MS-P3* on LG6b (2014) (Fig 1 and Table 1). *MS-P1* was detected on both maternal and paternal maps at almost the same locus as that detected on the integrated map (data not shown).

In the IM analysis, significant QTLs at *p* = 0.05 for the APF were detected at *MS-F1* on LG6a (2014–2016 and the average for the three years) (S4 Table). In addition, the MQM analysis of those loci confirmed significant QTLs at *p* = 0.1 at *MS-F1* on LG6a (2014–2016 and the average for the three years) and detected *MS-F2* on LG8 (2015) (Fig 1 and Table 1). *MS-F1* was detected on both the maternal and paternal maps at nearly the same locus as that on the integrated map (data not shown). *MS-P1* was detected at nearly the same locus in 2014–2016 and the average for the three years; it had the highest value of the LOD score and the percentage of variance explained of the detected QTLs. Similar results were obtained for *MS-F1* (S4 Table and Table 1). These significant QTLs were also detected by the KW test (Table 1). Therefore, we focused on *MS-P1* and *MS-F1* in the following analysis.

### Associations between alleles located in the vicinity of *MS-P1* and *MS-F1* and phenotypes in F_1_ population of O46-O56

The associations between the detected QTLs and alleles of the neighboring SSR markers located close to *MS-P1* and *MS-F1* were examined using the average data for the three years from the O46-O56 population. *MS-P1* was located in the range of 5.1 cM between TSRF161, GSR5112, NSX161, and NSX132. *MS-F1* was located in the range of 1.8 cM between TSRA107 and SSR08B32 (Fig 1). TSRF161, GSR5112, TSRA107, and SSR08B32 were used further in association analysis. The genotypes at the marker sites in ‘Okitsu No. 46’ and ‘Okitsu No. 56’ are shown in S5 Table.

**Fig 2:**
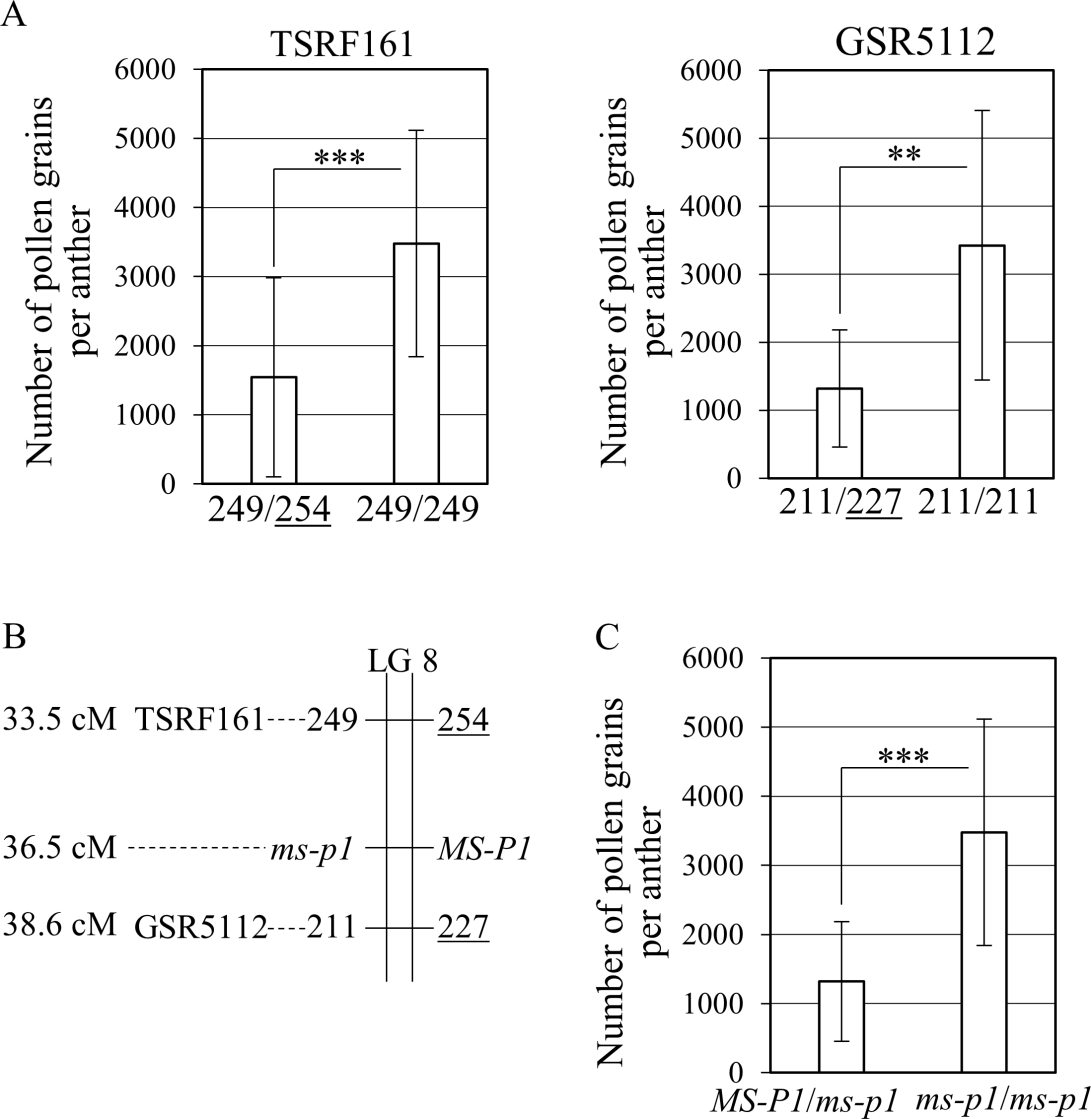
Associations between the number of pollen grains per anther and *MS-P1*. The number of pollen grains per anther is examined in ‘Okitsu No. 46’ × ‘Okitsu No. 56’ (O46-O56) population and it is based on the average for three years. (A) Associations between the number of pollen grains per anther and the alleles of TSRF161 and GSR5112. Average of the number of pollen grains per anther in 8–17 seedlings is shown. (B) Enlarged genetic linkage map of O46-O56 near *MS-P1*. The loci with the highest logarithm of the odds score within the range of *MS-P1* and the flanking markers are shown. The alleles linked with *MS-P1* and *ms-p1* are shown on the right side and left side of LG8, respectively. (C) Selection efficacy of the *MS-P1* haplotype block in the O46-O56 population. *MS-P1*/*ms-p1* shows individuals with both “254” allele at TSRF161 and “227” allele at GSR5112 (*n* = 15). *ms-p1*/*ms-p1* indicates individuals without both the “254” and the “227” allele (*n* = 8). *MS-P1* and *ms-p1* indicate a quantitative trait locus (QTL) for the number of pollen grains per anther. Underlines indicate alleles linked with *MS-P1* and derived from ‘Okitsu No. 46’. Bars indicate standard deviation. Asterisks (** and ***) show significance levels at *p* < 0.05 and 0.01, respectively.

Initially, we validated the association between the alleles at the markers near *MS-P1* and the NPG (Fig 2). The NPG in individuals with “254” allele derived from ‘Okitsu No. 46’ at TSRF161 was significantly lower than the NPG in individuals lacking the “254” allele (Fig 2A). Similarly, the average NPG in individuals that had the “227” allele derived from ‘Okitsu No. 46’ at GSR5112 was significantly lower compared with other individuals lacking the “227” allele (Fig 2A). The linkage analysis indicated that the “254” allele at TSRF161 and the “227” allele at GSR5112 located on the same linkage phase in O46-O56 (Fig 2B). These results suggest that *MS-P1* locates on a haplotype block harboring the “254” allele at TSRF161 and the “227” allele at GSR5112 (Fig 2B).

The assumed haplotype block with *MS-P1* was verified by comparing the *MS-P1*/*ms-p1* line to the *ms-p1*/*ms-p1* line, where *MS-P1* is a dominant and *ms-p1* is a recessive haplotype block for reduced NPG (Fig 2B and 2C). The average NPG of individuals harboring *MS-P1*/*ms-p1* was significantly lower than that in *ms-p1*/*ms-p1* (Fig 2C). Additionally, the average NPG of individuals harboring *MS-P1*/*ms-p1* was 1,321, which was comparable to the NPG in Satsuma (S1 Fig). These results indicated that *MS-P1* locates on the haplotype block that contains both the “254” allele at TSRF161 and the “227” allele at GSR5112 and reduces the NPG.

**Fig 3:**
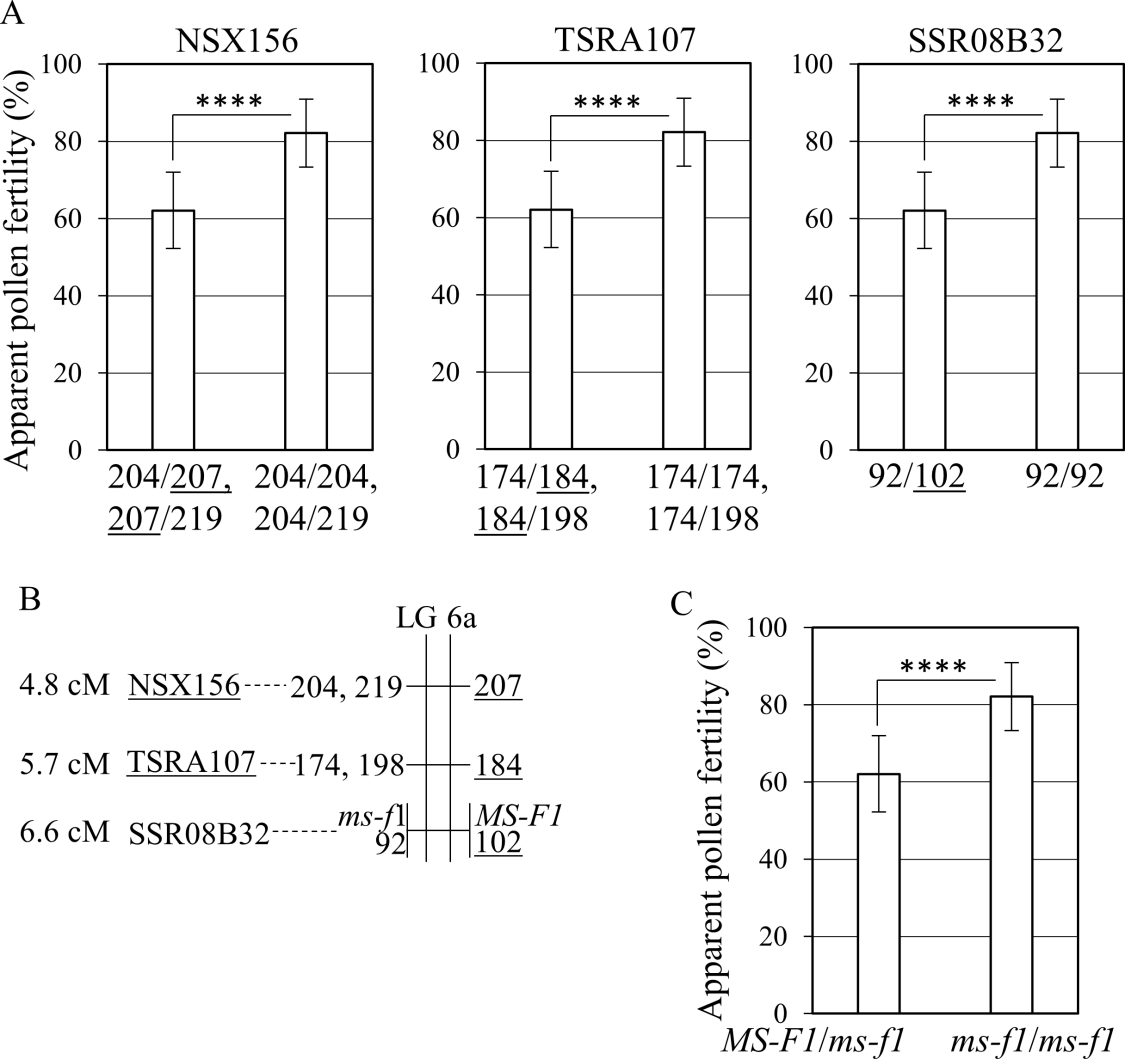
Association between apparent pollen fertility and *MS-F1*. The apparent pollen fertility is examined in ‘Okitsu No. 46’ × ‘Okitsu No. 56’ (O46-O56) population and it is based on the average of three years. (A) Association between apparent pollen fertility and alleles of NSX156, TSRA107, and SSR08B32. Average of apparent pollen fertility in 8–17 seedlings is shown. (B) Enlarged genetic linkage maps for O46-O56 near *MS-F1*. The loci with the highest logarithm of the odds score in the range of *MS-F1* and the flanking markers are shown. The alleles linked with *MS-F1* and *ms-f1* are shown on the right side and left side of LG6a, respectively. (C) Selection efficacy of the *MS-F1* haplotype block in the O46-O56 population. *MS-F1*/*ms-f1* shows individuals with both “207” allele at NSX 156 and “102” allele at SSR08B32 (*n* = 8). *ms-f1*/*ms-f1* indicates individuals without both the “207” and the “102” allele (*n* = 17). Underlines indicate the alleles linked with *MS-F1* and derived from ‘Okitsu No. 46’. Asterisk (****) shows significance level at *p* < 0.0001. Bars indicate standard deviation.

Next, we validated the association between the alleles at markers near *MS-F1* and the APF (Fig 3). The APF in individuals with the “207” allele derived from ‘Okitsu No. 46’ at NSX156 was significantly lower than it was in other individuals (Fig 3A). Similarly, the average APF in each individual containing the “184” allele at TSRA107 or “102” allele at SSR08B32 derived from ‘Okitsu No. 46’ was significantly lower compared with that in other individuals (Fig 3A). The linkage analysis indicated that the “207” allele at NSX156, “184” allele at TSRA107, and “102” allele at SSR08B32 located on the same linkage phase in O46-O56 (Fig 3B). These results suggest that *MS-F1* locates on the haplotype block harboring the “207” allele at NSX156, the “184” allele at TSRA107, and the “102” allele at SSR08B32 (Fig 3B).

The postulated haplotype block harboring *MS-F1* was verified by comparing *MS-F1*/*ms-f1* lines with *ms-p1*/*ms-p1* lines, where *MS-F1* was a dominant and *ms-f1* a recessive haplotype block for decreased APF (Fig 3B and 3C). APF was significantly lower in *MS-F1*/*ms-f1* than in *ms-p1*/*ms-p1* (Fig 3C), while the average APF in *MS-F1*/*ms-f1* was 62% and comparable to the levels detected in ‘Okitsu No. 46’ (S1 Fig). These results showed that *MS-F1* locates on the haplotype block that harbors both the “207” allele at NSX156 and “102” allele at SSR08B32 and decreases the APF.

### Validation of the effect of *MS-P1* and *MS-F1* in F_1_ population of O46-K

We validated the effect of the haplotype blocks containing *MS-P1* (*MS-P1* haplotype block) and *MS-F1* (*MS-F1* haplotype block) in 34 seedlings of O46-K (S1 Table). The genotypes at TSRF161 and GSR5112 in ‘Kara’ are 246/254 and 211/227, respectively (S5 Table). The NPG in individuals that had the “254” allele derived from ‘Okitsu No. 46’, which linked to *MS-P1* and was derived from ‘Kara’ at TSRF161, was significantly lower compared with the NPG in other individuals (S3A Fig). Similarly, the NPG was significantly lower in individuals with the “227” allele derived from ‘Okitsu No. 46’ and ‘Kara’ at GSR5112 than in other individuals (S3A Fig). Both ‘Okitsu No. 46’ and ‘Kara’ were heterozygous for the “254” allele at TSRF161 and “227” allele at GSR5112 (S5 Table), and their crossing produced individuals that were homozygous for “254” allele at TSRF161 or “227” allele at GSR5112. The average NPG in individuals that were homozygous for those alleles tended to be lower when compared with those of individuals that were heterozygous for the same alleles, although the results were collected for only one year (data not shown). We validated the efficacy of the *MS-P1* haplotype block by comparing the NPG in the *MS-P1*/*ms-p1* and *ms-p1*/*ms-p1* lines during the two years. The average NPG was significantly lower in *MS-P1*/*ms-p1* than in *ms-p1*/*ms-p1* (S3B Fig). Additionally, the average NPG in *MS-P1*/*ms-p1* was 823, which was comparable to the NPG in Satsuma (S1 Fig). These results showed that the reduced NPG in O46-K was caused not only by the *MS-P1* haplotype block in ‘Okitsu No. 46’ but also by the “254” allele at TSRF161 and “227” allele at GSR5112 in ‘Kara’. Furthermore, the results suggest that individuals with a trait for reduced NPG can be screened by using the flanking markers of *MS-P1*.

Next, we validated the effect of the *MS-F1* haplotype block. The genotypes at NSX156, TSRA107, and SSR08B32 in ‘Kara’ are shown in S5 Table. The APF in individuals with the “207” allele at NSX156 derived from ‘Okitsu No. 46’ was significantly lower compared with that in other individuals (S3C Fig). Similarly, the individuals containing the “184” allele at TSRA107 or “102” allele at SSR08B32 derived from ‘Okitsu No. 46’ had an APF significantly lower compared with that in other individuals (S3C Fig). The average APF in *MS-F1*/*ms-f1* (65%) was significantly lower compared with that in *ms-f1*/*ms-f1* (S3D Fig) and comparable to the APF of ‘Okitsu No. 46’ (S1 Fig). These results confirmed that the *MS-F1* haplotype block decreases the APF in O46-K and the flanking markers of *MS-F1* are suitable for screening of individuals with low APF.

### Origin of NPG and APF inferred by pedigree analysis

**Fig 4:**
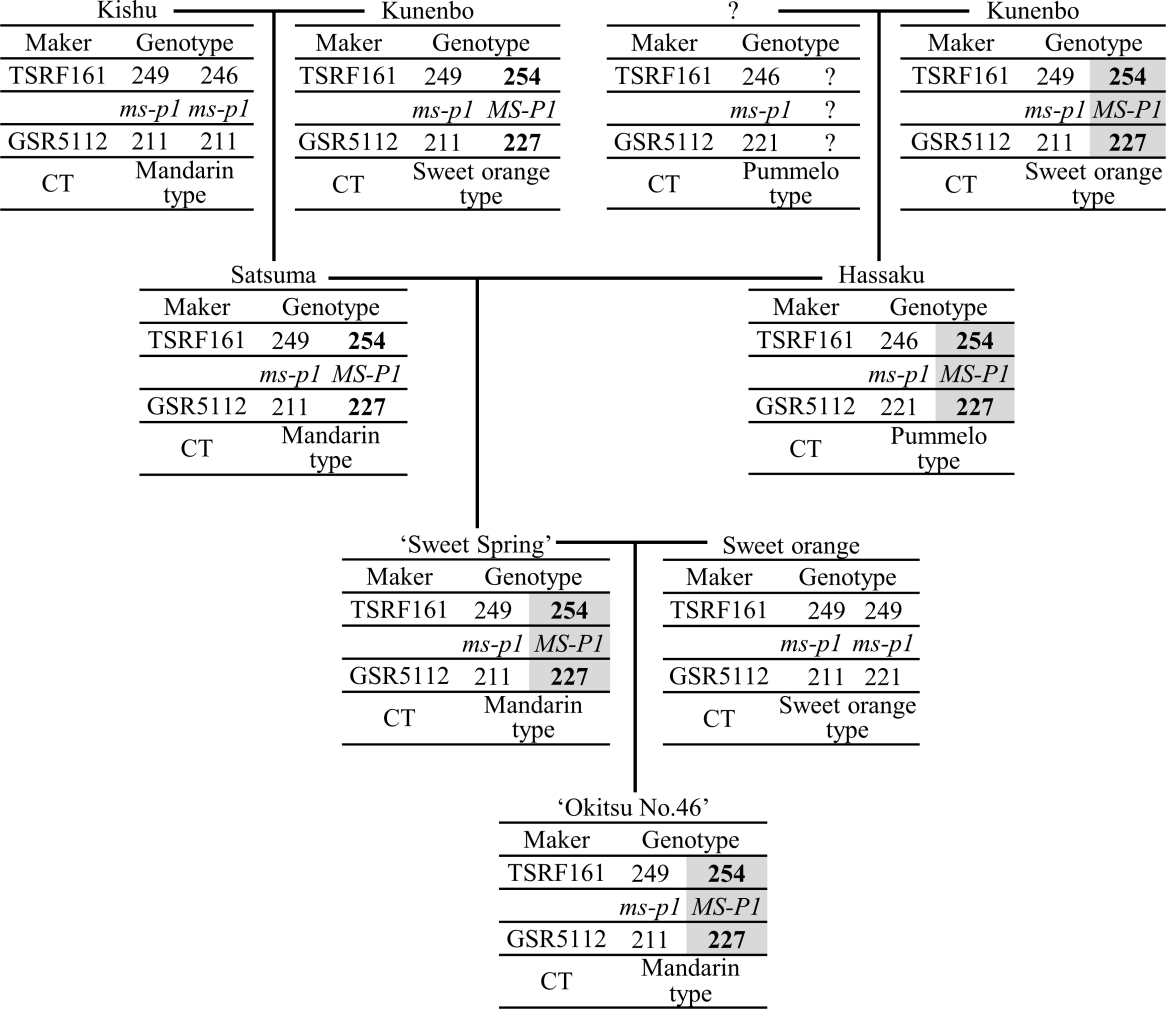
Inheritance of the alleles at TSRF161 and GSR5112 in the pedigree of ‘Okitsu No. 46’. Genotypes of simple sequence repeat markers and classes of organelle genotype (CT) were sourced from a previous report (Shimizu et al. 2016). *MS-P1* indicates the position of the quantitative trait locus for the number of pollen grains per anther. The alleles assumed to be derived from kunenbo are indicated in bold. The transmission pattern of the *MS-P1* haplotype block in ‘Okitsu No. 46’ is indicated in gray.

The origin of the traits of reduced NPG and decreased APF were estimated by focusing on the inheritance of each *MS-P1* haplotype block and *MS-F1* haplotype block derived from ‘Okitsu No. 46’. The seed parent of ‘Okitsu No. 46’ is ‘Sweet Spring’ and its pollen parent is sweet orange ‘Trovita’. The seed parent of ‘Sweet Spring’ is Satsuma and its pollen parent is hassaku [31,33]. Shimizu et al. revealed that hassaku is a selection from a cross of an unknown seed parent and kunenbo as the pollen parent [18]. Likewise, recent studies revealed that the pollen parent of Satsuma is kunenbo and the seed parent is Kishu [18,34]. Additionally, kunenbo and Kishu produce more pollen grains per anther than does Satsuma (S1 Fig). The derivation of the *MS-P1* haplotype block in ‘Okitsu No. 46’ was assessed by tracing the inheritance of the “254” allele at TSRF161 and “227” allele at GSR5112 in ancestral cultivars of ‘Okitsu No. 46’ (Fig 4). The pedigree analysis revealed that the *MS-P1* haplotype block of ‘Okitsu No. 46’ was derived from kunenbo through ‘Sweet Spring’ and hassaku (Fig 4).

Satsuma also contained the *MS-P1* haplotype block (Fig 4), and the same haplotype block was expected in ‘Kara’ (S3 Fig). This result suggests that the *MS-P1* haplotype block in ‘Kara’ is derived from kunenbo. The pedigree analysis of the haplotype block with the “254” allele at TSRF161 and the “227” allele at GSR5112 in ‘Kara’ suggested that the “254” allele at TSRF161 was derived from kunenbo. The origin of the “227” allele at GSR5112 could not be inferred because the genotype at GSR5112 was identical in ‘King’ and Satsuma (S4 Fig).

**Fig 5:**
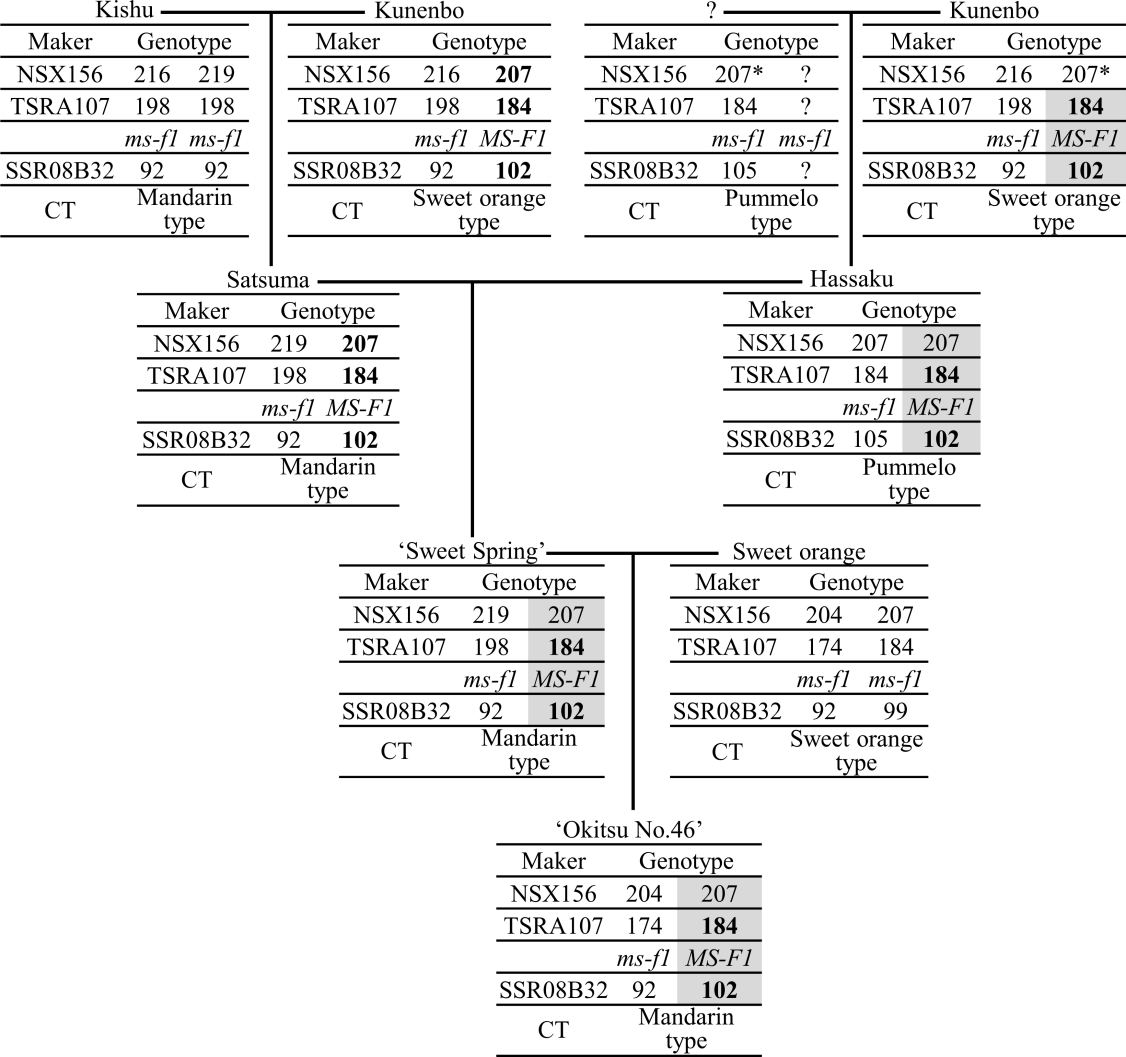
Inheritance of the alleles at NSX156, TRA107, and SSR08B32 in ancestors of ‘Okitsu No. 46’. Genotypes of simple sequence repeat markers and classes of organelle genotype (CT) were sourced from a previous report (Shimizu et al. 2016). The alleles assumed to be derived from kunenbo are indicated in bold. *MS-F1* indicates the position of quantitative trait loci for apparent pollen fertility. The transmission pattern of the *MS-F1* haplotype block in ‘Okitsu No. 46’ is indicated in gray.

The origin of the *MS-F1* haplotype block in ‘Okitsu No. 46’ was inferred by tracing the inheritance of the “207” allele at NSX156 and the “184” allele at TSRA107 in ancestral cultivars of ‘Okitsu No. 46’ (Fig 5). The pedigree analysis failed to identify the origin of the “207” allele at NSX156, while the *MS-F1* haplotype block was derived from kunenbo through ‘Sweet Spring’ and hassaku by tracing the inheritance of the “184” allele at TSRA107 and “102” allele at SSR08B32. This haplotype block was also inferred for Satsuma (Fig 5).

## Discussion

It has been proposed that male sterility derived from Satsuma is caused by cooperative action of the cytoplasm derived from Satsuma and nuclear genes [4–6], whereas the locus suitable for MAS of male sterility in citrus breeding has not been identified. In the present study, we identified three QTLs (*MS-P1*, *2*, *3*) for the NPG and two QTLs (*MS-F1*, *2*) for APF by analyzing the F_1_ population of O46-O56 (Fig 1 and Table 1). The associations between the NPG and the allele linked to *MS-P1* and between the APF and the allele linked to *MS-F1* was clarified (Figs 2 and 3). The *MS-P1* haplotype block indicated the association with reduced NPG and the *MS-F1* haplotype block decreased the APF in the F_1_ population of O46-K. The pedigree analysis identified the origin of both *MS-P1* and *MS-F1* as kunenbo (Figs 4 and 5).

### Criterion of male sterility

Although the NPG in Satsuma was 1,320 and that in ‘Okitsu No.46’ was 639, pollen grains were not observed on the outside of their anthers (S1 and S2 Figs). In contrast, the NPG in ‘Okitsu No. 56’ and ‘Kara’ was approximately 5,000 and the outside of their anthers was covered with many pollen grains (S1 and S2 Figs). These results suggest that the release of pollen grains from anthers depends on the NPG. As the pollen grains in Satsuma were not released from the anthers, a major criterion of male sterility is assumed to be the presence of less than approximately 1,300 pollen grains per anther. In addition, male sterility is assumed to be determined by APF as a secondary cause in the case of more than 1,300 pollen grains per anther.

In the present study, the NPG of 1,320 in *MS-P1/ms-p1* and APF of 62% in *MS-F1/ms-f1* in O46-O56 (Figs 2 and 3), as well as that similar results were observed in O46-K (S2 Fig), suggests that seedlings with both *MS-P1* and *MS-F1* haplotype blocks have severe male sterility.

### Derivation of male sterility in Satsuma progenies

Several reports suggested the involvement of nuclear genes in male sterility [5,35–38]. Guo et al. [7,8] and Zheng et al. [11] have tried to induce male sterility by replacing the cytoplasm by that of Satsuma type with cell fusion in Hirado buntan and Zheng et al. observed aborted pollen grains in the regenerated plants [13]. Zhang et al. [13] suggests that male sterility is caused by cooperative action of the cytoplasm derived from Satsuma and nuclear genes. Our recent study also demonstrated the involvement of nuclear genes in the offspring of Satsuma and possible contribution of cytoplasm in the expression of male sterility [14]. In the present study, major QTLs for NPG and APF (*MS-P1* and *MS-F1*) were stably detected in 2014–2016 and in the average for the three years in F_1_ population of O46-O56 (Figs 1–3 and Table 1), and their effect was observed in F_1_ population of O46-K (S3 Fig). These results strongly suggest that nuclear genes involved in male sterility are derived from Satsuma.

Recent studies revealed Kishu as the seed parent of Satsuma [18,34], suggesting that male sterility derived from Satsuma is likely caused by cooperative action of the cytoplasm derived from Kishu and nuclear genes. The observed NPG was low in ‘Okitsu No. 46’, Satsuma, and ‘Sweet Spring’, but high in kunenbo and hassaku (S1 Fig). All of these cultivars contained the *MS-P1* haplotype block (Fig 4 and S1 Fig). As the cytoplasm of ‘Okitsu No. 46’, ‘Sweet Spring’, and Satsuma is of the ‘mandarin’ type derived from Kishu, the cytoplasm of kunenbo is of the ‘Sweet Orange’ type, and the cytoplasm of hassaku is the pummelo type (Fig 4) [18], the reduced NPG trait would be expressed only in the presence of both the *MS-P1* haplotype block and the cytoplasm derived from Kishu.

The cytoplasm of ‘Okitsu No. 56’ is of the ‘mandarin’ type [18] derived from Kishu, but both ‘Okitsu No.56’ and Kishu lacked the *MS-P1* haplotype block (Fig 4). Their observed NPG was approximately 4,000, which was greater than that of ‘Okitsu No. 46’, ‘Sweet Spring’, and Satsuma, but lower than that in kunenbo and hassaku (S1 Fig). The *MS-P1* haplotype block is present in ‘Okitsu No. 46’, ‘Sweet Spring’, Satsuma, kunenbo, and hassaku, but only the former three cultivars have the ‘mandarin’ type cytoplasm derived from Kishu. These results strongly suggest that reduced NPG trait is expressed only in the presence of both the *MS-P1* haplotype block and the cytoplasm derived from Kishu, although some reduction in NPG is possible by only having the cytoplasm derived from Kishu.

Similarly, the APF was low in Satsuma, ‘Sweet Spring’, and ‘Okitsu No. 46’, all of which are inferred to have both the *MS-F1* haplotype block and the cytoplasm derived from Kishu. In contrast, the APF was high in kunenbo and hassaku, whose cytoplasm did not originate from Kishu and both were inferred to have the *MS-F1* haplotype block (Fig 5 and S1 Fig). The APF was also high in ‘Okitsu No. 56’ and Kishu, with cytoplasm originating from Kishu (Fig 5 and S1 Fig). These results indicate that decreased APF trait is expressed only in the presence of both the *MS-F1* haplotype block and the cytoplasm derived from Kishu.

The discrepancies between the observed phenotype and the genotypes in hassaku and kunenbo imply a contribution of another unidentified gene to male fertility when the involvement of their cytoplasm is not considered. However, when taking into account the contribution of cytoplasm in the expression of male sterility, a combination of a nuclear gene and a particular cytoplasm can explain the observed male sterility in the offspring of Satsuma without postulating an unidentified gene.

Yamamoto et al. suggested the cooperative action of the cytoplasm derived from Satsuma and nuclear genes by reciprocal crosses [5]. Given the Yamamoto’s report, the above results suggest that the expression of male sterility requires the presence of cytoplasm derived from Satsuma (Kishu). Iwamasa proposed that the nuclear factor that regulates male sterility in the progenies of Satsuma, including ‘Kiyomi’, is derived from Satsuma [1]. The present study suggested that the nuclear factor that regulates male sterility derived from Satsuma originated from kunenbo. Two possibilities of a mutation (in a cytoplasmic gene or in *MS-P1* and *MS-F1*) that may occur in Satsuma could result in male sterility of its progeny. However, the two possibilities seem less feasible for the following reasons. In the event of cytoplasmic mutation, it is unlikely that all mitochondria genome were replaced to hold mutated gene even if mutation happened in a mitochondrial gene. Regarding the latter possibility, the mutation in *MS-P1* and *MS-F1* that occurred during vegetative propagation of Satsuma trees or when Satsuma was selected from the cross between Kishu and kunenbo contradicts the findings that *MS-P1* and *MS-F1* in ‘Sweet Spring’ and ‘Okitsu No.46’ were derived not from Satsuma but from kunenbo.

### The application of *MS-P1* and *MS-F1* in breeding

The seedlings containing the *MS-P1* haplotype block in the F_1_ population of O46-K correlated with those with reduced NPG trait (S3B Fig). Similarly, the seedlings containing the *MS-F1* haplotype block were correlated with those with decreased APF trait (S3D Fig). Thus, the selection for these haplotype blocks will ensure male sterility not only in F_1_ population of O46-O56 but also in other F_1_ populations in which ‘Okitsu No. 46’ is used as a seed parent. The deduced large contribution of *MS-P1* to the phenotypic variation (37.7% for the average of 2014–2016) supported the availability of this locus in selecting male sterile seedlings. Although the data were obtained only for 2015, the NPG in individuals homozygous for “254” at TSRF161 or “227” at GSR5112 was less than that in Satsuma in an F_1_ population of O46-K in 2015 (data not shown); the homozygous individuals did not bloom in 2014. The “254” allele in ‘Kara’, similar to that in ‘Okitsu No. 46’, was inferred to be derived from kunenbo (S4 Fig and Fig 4). These results suggest that the “254” allele at TSRF161 is linked to the *MS-P1* haplotype block and strongly affects the NPG reduction. Thus, a very low NPG can be obtained by selecting individuals homozygous for “254” at TSRF161 that are derived from kunenbo.

‘Kara’ has the “254” allele at TSRF161 derived from kunenbo and the cytoplasm that originates from Kishu (S4 Fig); the NPG in ‘Kara’ is comparable with that in ‘Okitsu No. 56’ [14] (S1 Fig). Therefore, the NPG is not reduced in some cases of genetic background, even in the presence of *MS-P1* haplotype block and the cytoplasm derived from Kishu.

The parentage analysis revealed that the *MS-P1* and *MS-F1* haplotype blocks were derived from kunenbo (Figs 4 and 5). Because kunenbo is the ancestor of several cultivars, those cultivars may have the “254” allele at TSRF161, “227” allele at GSR5112, “184” allele at TSRA107, and “102” allele at SSR08B32 [18], and consequently the *MS-P1* and *MS-F1* haplotype blocks.

## Conclusions

In the present study, two QTLs were detected, *MS-P1*, which is a major QTL for reducing the NPG, and *MS-F1*, a major QTL for decreasing APF. Additionally, the involvement of nuclear genes in male sterility was confirmed. Reduced NPG trait requires not only the presence of the *MS-P1* haplotype block, but also a cytoplasm that is derived from Kishu. Similarly, decreased APF trait entails the presence of the *MS-F1* haplotype block and cytoplasm derived from Kishu. In addition, the *MS-P1* and *MS-F1* haplotype blocks that were found in several cultivars were inferred to be derived from kunenbo. Therefore, we expect that male sterile seedlings can be selected by using adjacent SSR markers linked to *MS-P1* and *MS-F1* haplotype blocks in cross populations of kunenbo progenies. However, these findings are based on two F_1_ populations and nine cultivars and further verification by using additional F_1_ populations and wide offspring cultivars of Satsuma is warranted. Thereby, they will not only be key to understanding the molecular mechanism of male sterility in citrus, but they will also contribute to the breeding of seedless citrus cultivars.

## Supporting information

S1 Fig Distribution of two traits that are the leading cause of male sterility observed among citrus cultivars and selections used in this study. (A) The number of pollen grains per anther and (B) apparent pollen fertility. Male sterility was evaluated based on the number of pollen grains per anther and apparent pollen fertility in 3–8 anthers per flower by staining with lactophenol blue. Each value represents the average of three flowers from the same tree. The evaluations were carried out in 2017. Columns with the same lowercase letter are not significantly different according to Tukey’s test (*p* < 0.05). Bars indicate standard deviation. Satsuma: the original tree of Satsuma.
(PDF)

S2 Fig Magnified view of the anthers in ‘Okitsu No. 46’, ‘Okitsu No.56’, and ‘Kara’. ‘Okitsu No. 46’, ‘Okitsu No.56’, and ‘Kara’ were used as parents of cross populations in this study. The anthers in Satsuma (the original tree of Satsuma) and hassaku are shown as controls. These anthers were collected after full bloom during anthesis on May 12, 2017. Bar = 2 mm.
(PDF)

S3 Fig Selection efficacy of *MS-P1* and *MS-F1* haplotype blocks in ‘Okitsu No. 46’ × ‘Kara’ population. The number of pollen grains per anther and apparent fertility represent the average for two years. (A) Association between the number of pollen grains per anther and the alleles of TSRF161 and GSR5112. Underlines indicate the alleles linked with *MS-P1* derived from ‘Okitsu No.46’. Average number of pollen grains per anther in 8–12 seedlings is shown. (B) Selection efficacy of *MS-P1* haplotype block in F_1_ population. *MS-P1*/*ms-p1* shows individuals containing both “254” allele at TSRF161 and “227” allele at GSR5112 (*n* = 10). *ms-p1*/*ms-p1* indicates individuals lacking those alleles (*n* = 8). (C) Association between apparent fertility and the alleles of NSX156, TSR107, and SSR08B32. Underlines indicate the alleles linked with *MS-F1* derived from ‘Okitsu No.46’. Average apparent pollen fertility in 4–12 seedlings is shown. (D) Selection efficacy of *MS-F1* haplotype block in F_1_ population. *MS-F1*/*ms-f1* shows individuals with both “184” allele at TSRA107 and “102” allele at SSR08B32 (*n* = 4). *ms-f1*/*ms-f1* indicates individuals lacking those alleles (*n* = 8). Asterisks (**, ***, ****) show significance levels at *p* < 0.05, 0.01, and 0.005, respectively.
(PDF)

S4 Fig Inheritance of the alleles of TSRF161 and GSR5112 linked with *MS-P1* in ‘Kara’ ancestors. Genotypes of simple sequence repeat markers and classes of organelle genotype (CT) were sourced from a previous report (Shimizu et al. 2016). The alleles derived from kunenbo are indicated in bold. *: Allele “227” at GSR5112 could not be estimated because the genotypes at GSR5112 were the same in Satsuma and King. CT: classes of organelle genotype.
(PDF)

S1 Table F_1_ populations used in this study. NPG: number of pollen grains per anther; APF: apparent pollen fertility. #: Pollen grains were not detected in five seedlings of the ‘Okitsu No.46’ × ‘Kara’ cross. Therefore, the number of seedlings used for investigation of the apparent pollen fertility was lower by five individuals than the number of seedlings used for assessing the number of pollen grains per anther.
(DOCX)

S2 Table DNA markers used in this study. (XLSX)

S3 Table The number of simple sequence repeat (SSR) markers, segregation type, mapped loci, and the total length of the constructed linkage map in ‘Okitsu No. 46’ × ‘Okitsu No. 56’ population. (DOCX)

S4 Table Quantitative trait loci (QTLs) for the number of pollen grains per anther (NPG) and apparent pollen fertility (APF) in ‘Okitsu No. 46’ × ‘Okitsu No. 56’ population detected by interval mapping for three consecutive years. QTLs represent the position of the highest logarithm of the odds (LOD) value. LG: linkage group; % Expl. indicates the percentage of phenotypic variation explained by QTL. Average: the average value obtained in 2014–2016.
(DOCX)

S5 Table Genotypes of the flanking markers for *MS-P1* and *MS-F1*. Underlines indicate the alleles linked to the quantitative trait loci (QTLs) *MS-P1* or *MS-F1*.
(DOCX)
