# Supplementary material for: QTL mapping of male sterility and transmission pattern in progeny of Satsuma mandarin
Source: PLoS One. 2018 Jul 17;13(7):e0200844. doi: 10.1371/journal.pone.0200844 (PMC6049952; doi:10.1371/journal.pone.0200844)
Supplement: S1 Fig — (A) The number of pollen grains per anther and (B) apparent pollen fertility. Male sterility was evaluated based on the number of pollen grains per anther and apparent pollen fertility in 3–8 anthers per flower by staining with lactophenol blue. Each value represents the average of three flowers from the same tree. The evaluations were carried out in 2017. Columns with the same lowercase letter are not significantly different according to Tukey’s test (p < 0.05). Bars indicate standard deviation. Satsuma: the original tree of Satsuma. (PDF) [file pone.0200844.s001.pdf]

A

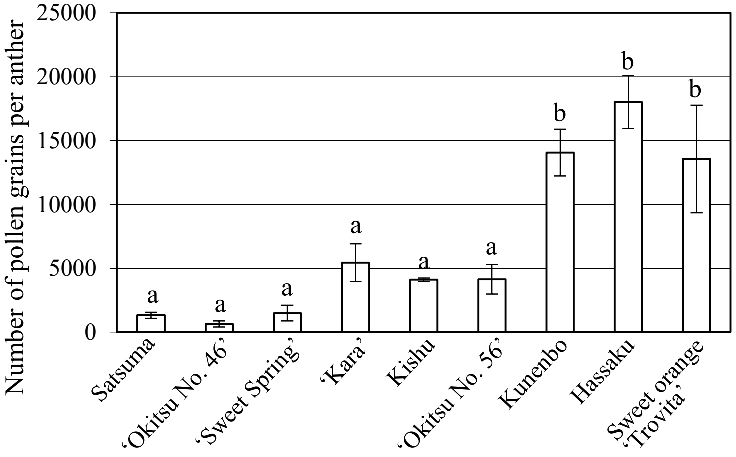

B

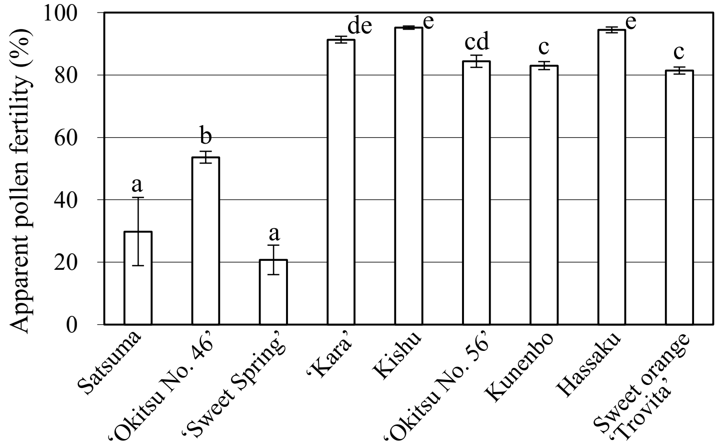

S1 Fig. Distribution of two traits that are the leading cause of male sterility observed among citrus cultivars and selections used in this study. (A) The number of pollen grains per anther and (B) apparent pollen fertility. Male sterility was evaluated based on the number of pollen grains per anther and apparent pollen fertility in 3–8 anthers per flower by staining with lactophenol blue. Each value represents the average of three flowers from the same tree. The evaluations were carried out in 2017. Columns with the same lowercase letter are not significantly different according to Tukey's test ($p < 0.05$). Bars indicate standard deviation. Satsuma: origin of Satsuma.
