# Supplementary material for: QTL mapping of male sterility and transmission pattern in progeny of Satsuma mandarin
Source: PLoS One. 2018 Jul 17;13(7):e0200844. doi: 10.1371/journal.pone.0200844 (PMC6049952; doi:10.1371/journal.pone.0200844)
Supplement: S2 Fig — ‘Okitsu No. 46’, ‘Okitsu No.56’, and ‘Kara’ were used as parents of cross populations in this study. The anthers in Satsuma (the original tree of Satsuma) and hassaku are shown as controls. These anthers were collected after full bloom during anthesis on May 12, 2017. Bar = 2 mm. (PDF) [file pone.0200844.s002.pdf]

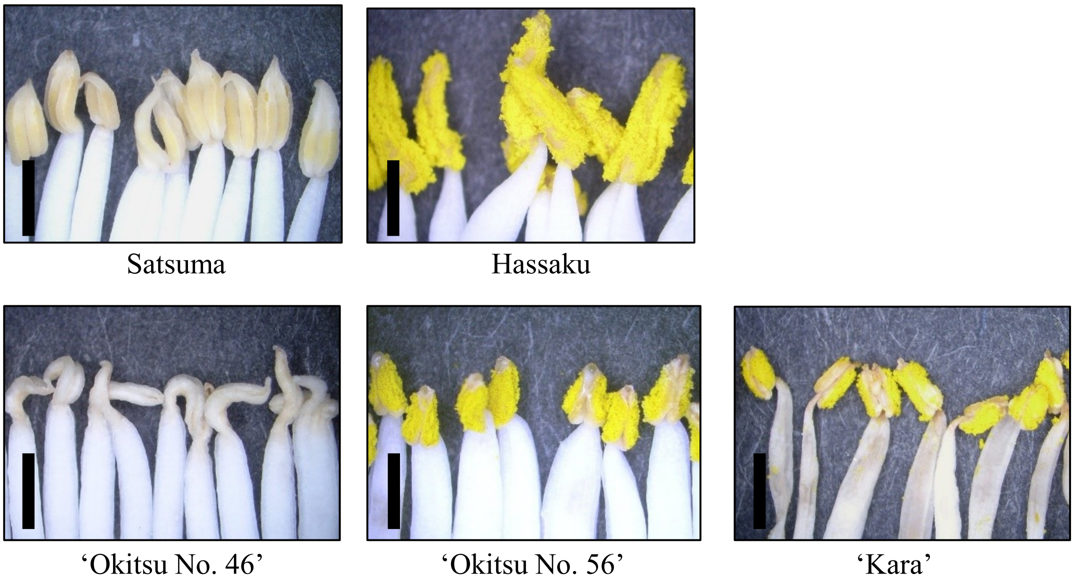

S2 Fig. Magnified view of the anthers in 'Okitsu No. 46', 'Okitsu No.56', and 'Kara'. 'Okitsu No. 46', 'Okitsu No.56', and 'Kara' were used as parents of cross populations in this study. The anthers in Satsuma (the original tree of Satsuma) and hassaku are shown as controls. These anthers were collected after full bloom during anthesis on May 12, 2017. Bar = 2 mm.
