# Supplementary material for: QTL mapping of male sterility and transmission pattern in progeny of Satsuma mandarin
Source: PLoS One. 2018 Jul 17;13(7):e0200844. doi: 10.1371/journal.pone.0200844 (PMC6049952; doi:10.1371/journal.pone.0200844)
Supplement: S3 Fig — The number of pollen grains per anther and apparent fertility represent the average for two years. (A) Association between the number of pollen grains per anther and the alleles of TSRF161 and GSR5112. Underlines indicate the alleles linked with MS-P1 derived from ‘Okitsu No.46’. Average number of pollen grains per anther in 8–12 seedlings is shown. (B) Selection efficacy of MS-P1 haplotype block in F1 population. MS-P1/ms-p1 shows individuals containing both “254” allele at TSRF161 and “227” allele at GSR5112 (n = 10). ms-p1/ms-p1 indicates individuals lacking those alleles (n = 8). (C) Association between apparent fertility and the alleles of NSX156, TSR107, and SSR08B32. Underlines indicate the alleles linked with MS-F1 derived from ‘Okitsu No.46’. Average apparent pollen fertility in 4–12 seedlings is shown. (D) Selection efficacy of MS-F1 haplotype block in F1 population. MS-F1/ms-f1 shows individuals with both “184” allele at TSRA107 and “102” allele at SSR08B32 (n = 4). ms-f1/ms-f1 indicates individuals lacking those alleles (n = 8). Asterisks (**, ***, ****) show significance levels at p < 0.05, 0.01, and 0.005, respectively. (PDF) [file pone.0200844.s003.pdf]

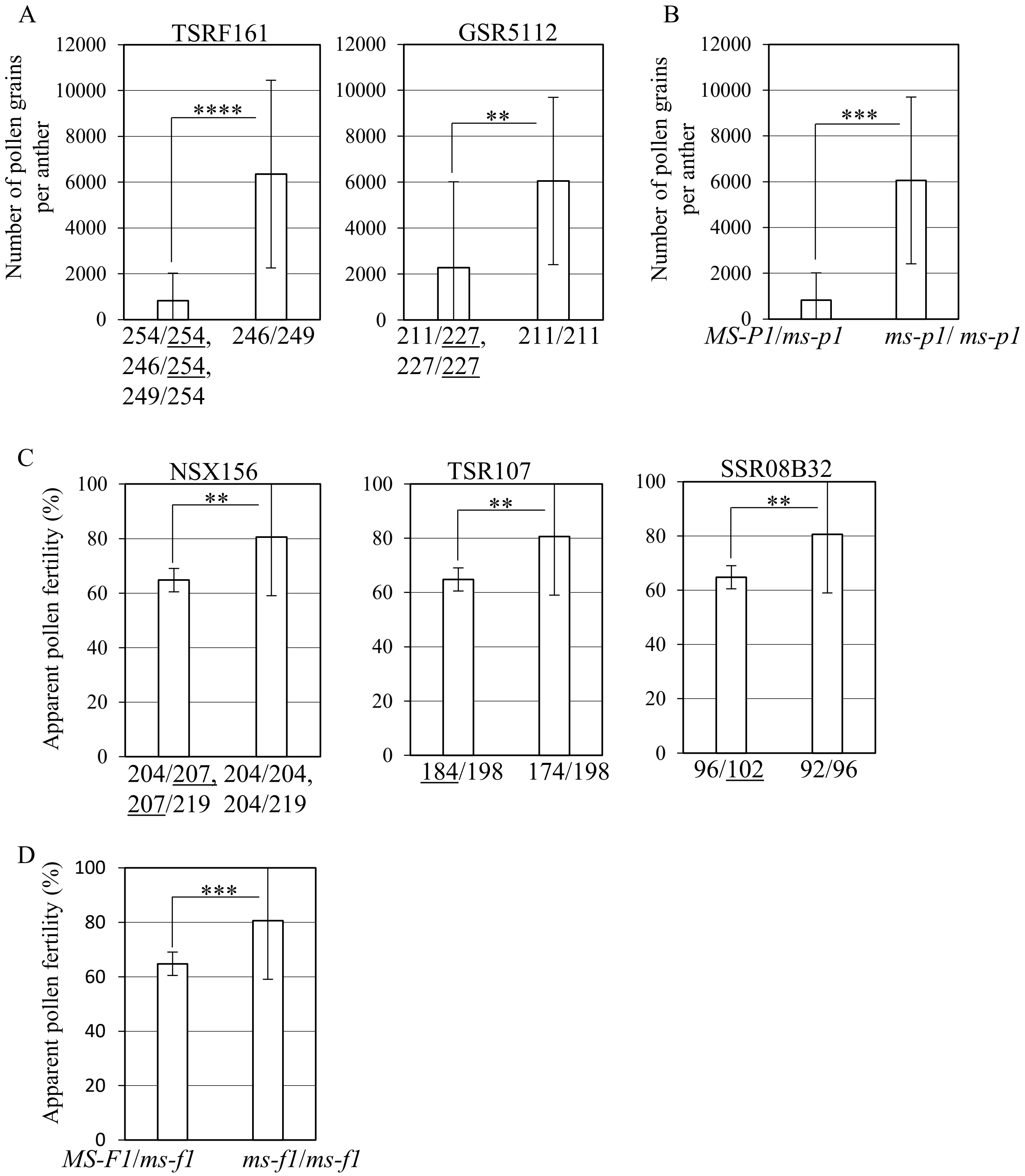

S3 Fig. Selection efficacy of *MS-P1* and *MS-F1* haplotype blocks in 'Okitsu No. 46' × 'Kara' population. The number of pollen grains per anther and apparent fertility represent the average for two years. (A) Association between the number of pollen grains per anther and the alleles of TSRF161 and GSR5112. Underlines indicate the alleles linked with *MS-P1* derived from 'Okitsu No.46'. Average number of pollen grains per anther in 8–12 seedlings is shown. (B) Selection efficacy of *MS-P1* haplotype block in F_1 population. *MS-P1/ms-p1* shows individuals containing both "254" allele at TSRF161 and "227" allele at GSR5112 (n = 10). *ms-p1/ms-p1* indicates individuals lacking those alleles (n = 8). (C) Association between apparent fertility and the alleles of NSX156, TSR107, and SSR08B32. Underlines indicate the alleles linked with *MS-F1* derived from 'Okitsu No.46'. Average apparent pollen fertility in 4–12 seedlings is shown. (D) Selection efficacy of *MS-F1* haplotype block in F_1 population. *MS-F1/ms-f1* shows individuals with both "184" allele at TSRA107 and "102" allele at SSR08B32 (n = 4). *ms-f1/ms-f1* indicates individuals lacking those alleles (n = 8). Asterisks (**, ***, ****) show significance levels at $p < 0.05$, 0.01, and 0.005, respectively.
