# Supplementary material for: QTL mapping of male sterility and transmission pattern in progeny of Satsuma mandarin
Source: PLoS One. 2018 Jul 17;13(7):e0200844. doi: 10.1371/journal.pone.0200844 (PMC6049952; doi:10.1371/journal.pone.0200844)
Supplement: S4 Fig — Genotypes of simple sequence repeat markers and classes of organelle genotype (CT) were sourced from a previous report (Shimizu et al. 2016). The alleles derived from kunenbo are indicated in bold. *: Allele “227” at GSR5112 could not be estimated because the genotypes at GSR5112 were the same in Satsuma and King. CT: classes of organelle genotype. (PDF) [file pone.0200844.s004.pdf]

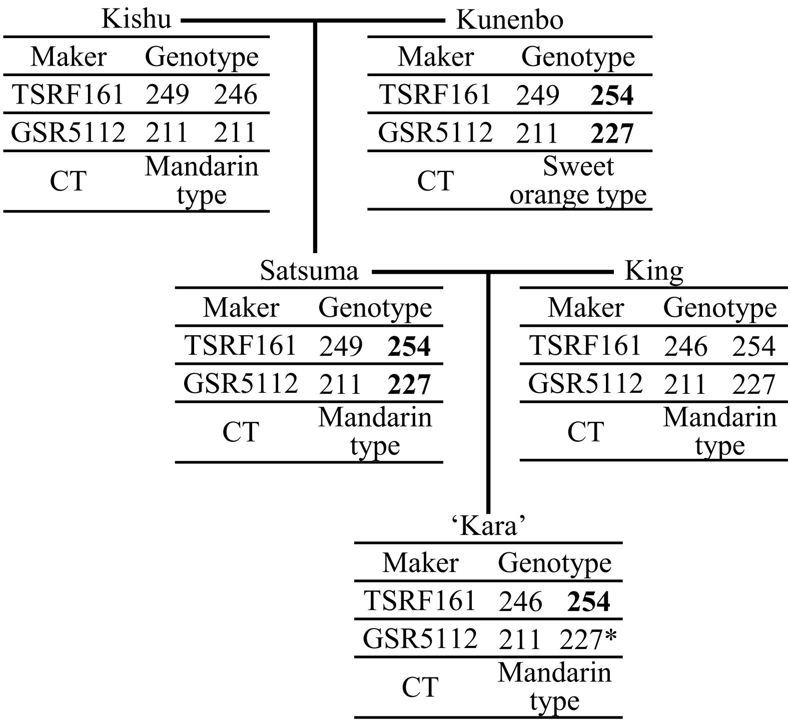

S4 Fig. Inheritance of the alleles of TSRF161 and GSR5112 linked with *MS-P1* in 'Kara' ancestors. Genotypes of simple sequence repeat markers and classes of organelle genotype (CT) were sourced from a previous report (Shimizu et al. 2016). The alleles derived from kunenbo are indicated in bold. *: Allele "227" at GSR5112 could not be estimated because the genotypes at GSR5112 were the same in Satsuma and King. CT: classes of organelle genotype.
