# Supplementary material for: QTL mapping of male sterility and transmission pattern in progeny of Satsuma mandarin
Source: PLoS One. 2018 Jul 17;13(7):e0200844. doi: 10.1371/journal.pone.0200844 (PMC6049952; doi:10.1371/journal.pone.0200844)
Supplement: S1 Table — NPG: number of pollen grains per anther; APF: apparent pollen fertility. #: Pollen grains were not detected in five seedlings of the ‘Okitsu No.46’ × ‘Kara’ cross. Therefore, the number of seedlings used for investigation of the apparent pollen fertility was lower by five individuals than the number of seedlings used for assessing the number of pollen grains per anther. (DOCX) [file pone.0200844.s005.docx]

|  |  | Number of seedlings | | | | | | | |
| --- | --- | --- | --- | --- | --- | --- | --- | --- | --- |
| Cross combination | Population size | For investigation of the NPG | | | | For investigation of APF | | | |
|  |  | 2014 | 2015 | 2016 | common | 2014 | 2015 | 2016 | common |
| ‘Okitsu No.46’ × ‘Okitsu No.56’ | 57 | 34 | 49 | 54 | 25 | 34 | 49 | 54 | 25 |
| ‘Okitsu No.46’ × ‘Kara’ | 34 | 21# | 33 | - | 20 | 16# | 33 | - | 16 |

**S1 Table. F_1_**  **Populations used in this study.**

NPG: number of pollen grains per anther; APF: apparent pollen fertility. #: Pollen grains were not detected in five seedlings of the ‘Okitsu No.46’ × ‘Kara’ cross. Therefore, the number of seedlings used for investigation of the apparent pollen fertility was lower by five individuals than the number of seedlings used for assessing the number of pollen grains per anther.

Goto, S. et al. QTL Mapping of Male Sterility and Transmission Pattern in Progeny of Satsuma Mandarin
