# Supplementary material for: QTL mapping of male sterility and transmission pattern in progeny of Satsuma mandarin
Source: PLoS One. 2018 Jul 17;13(7):e0200844. doi: 10.1371/journal.pone.0200844 (PMC6049952; doi:10.1371/journal.pone.0200844)
Supplement: S3 Table — (DOCX) [file pone.0200844.s007.docx]

**S3 Table. The number of simple sequence repeat (SSR) markers, segregation type, mapped loci, and the total length of the constructed linkage map in ‘Okitsu No. 46’ × ‘Okitsu No. 56’ population.**

| Segregation type | | Mapped loci |
| --- | --- | --- |
|  | <nnxnp> | 56 |
|  | <lmxll> | 98 |
|  | <hkxhk> | 34 |
|  | <efxeg> | 39 |
|  | <abxcd> | 8 |
| Total loci | | 235 |
| Total length (cM) | | 758.5 |

Goto, S. et al. QTL Mapping of Male Sterility and Transmission Pattern in Progeny of Satsuma Mandarin
