# Supplementary material for: QTL mapping of male sterility and transmission pattern in progeny of Satsuma mandarin
Source: PLoS One. 2018 Jul 17;13(7):e0200844. doi: 10.1371/journal.pone.0200844 (PMC6049952; doi:10.1371/journal.pone.0200844)
Supplement: S4 Table — QTLs represent the position of the highest logarithm of the odds (LOD) value. LG: linkage group; % Expl. indicates the percentage of phenotypic variation explained by QTL. Average: the average value obtained in 2014–2016. (DOCX) [file pone.0200844.s008.docx]

**S4 T****able. Quantitative trait loci (QTLs) for the number of pollen grains per anther (NPG) and apparent pollen fertility (APF) in ‘Okitsu No. 46’ × ‘Okitsu No. 56’ population detected by interval mapping for three consecutive years.**

| Traits | QTL | Data | LG | Position (cM) | LOD | % Expl. | Nearest marker |
| --- | --- | --- | --- | --- | --- | --- | --- |
| NPG | *MS-P1* | 2014 | 8 | 40.6 | 5.92 | 55.1 | GSR5112, NSX161, NSX132 |
|  |  | 2015 | 8 | 37.5 | 5.00 | 37.5 |  |
|  |  | Average for two years | 8 | 37.5-38.5 | 4.38 | 54.0 |  |
|  | *MS-P2* | 2014 | - | - | - | - | - |
|  |  | 2015 | 6b | 3.5-4.7 | 4.11 | 32.0 | SSR11A06, TSRB27 |
|  |  | Average for two years | - | - | n.d. | - | - |
|  | *MS-P3* | 2014 | - | - | n.d | - | - |
|  |  | 2015 | - | - | n.d. | - | - |
|  |  | Average for two years | 6b | 17.5 | 4.64 | 56.0 | CX0020 |
| APF | *MS-F1* | 2014 | 6a | 12.3-13.3 | 5.37 | 51.7 | TSRA107 |
|  |  | 2015 | 6a | 5.7 | 6.93 | 47.9 |  |
|  |  | Average for two years | 6a | 14.3 | 6.42 | 67.9 |  |

QTLs represent the position of the highest logarithm of the odds (LOD) value. LG: linkage group; % Expl. indicates the percentage of phenotypic variation explained by QTL. Average: the average value obtained in 2014–2016.

Goto, S. et al. QTL Mapping of Male Sterility and Transmission Pattern in Progeny of Satsuma Mandarin
