# Supplementary material for: QTL mapping of male sterility and transmission pattern in progeny of Satsuma mandarin
Source: PLoS One. 2018 Jul 17;13(7):e0200844. doi: 10.1371/journal.pone.0200844 (PMC6049952; doi:10.1371/journal.pone.0200844)
Supplement: S5 Table — Underlines indicate the alleles linked to the quantitative trait loci (QTLs) MS-P1 or MS-F1. (DOCX) [file pone.0200844.s009.docx]

**S5 Table. Genotypes of the flanking markers for *MS-P1* and *MS-F1*.**

| QTL | Flanking marker | ‘Okitsu No. 46’ | ‘Okitsu No. 56’ | ‘Kara’ |
| --- | --- | --- | --- | --- |
| *MS-P1* | TSRF161 | 249/254 | 249/249 | 246/254 |
|  | GSR5112 | 211/227 | 211/211 | 211/227 |
| *MS-F1* | NSX156 | 204/207 | 204/219 | 204/219 |
|  | TSRA107 | 174/184 | 174/198 | 198/198 |
|  | SSR08B32 | 92/102 | 92/92 | 92/96 |

Underlines indicate the alleles linked to the quantitative trait loci (QTLs) *MS-P1* or
*MS-F1*.

Goto, S. et al. QTL Mapping of Male Sterility and Transmission Pattern in Progeny of Satsuma Mandarin
